# Lower Termite Associations with Microbes: Synergy, Protection, and Interplay

**DOI:** 10.3389/fmicb.2016.00422

**Published:** 2016-04-08

**Authors:** Brittany F. Peterson, Michael E. Scharf

**Affiliations:** Department of Entomology, Purdue University, West LafayetteIN, USA

**Keywords:** termite, digestion, immunity, insect–microbe interactions, social insect, symbiosis

## Abstract

Lower-termites are one of the best studied symbiotic systems in insects. Their ability to feed on a nitrogen-poor, wood-based diet with help from symbiotic microbes has been under investigation for almost a century. A unique microbial consortium living in the guts of lower termites is essential for wood-feeding. Host and symbiont cellulolytic enzymes synergize each other in the termite gut to increase digestive efficiency. Because of their critical role in digestion, gut microbiota are driving forces in all aspects of termite biology. Social living also comes with risks for termites. The combination of group living and a microbe-rich habitat makes termites potentially vulnerable to pathogenic infections. However, the use of entomopathogens for termite control has been largely unsuccessful. One mechanism for this failure may be symbiotic collaboration; i.e., one of the very reasons termites have thrived in the first place. Symbiont contributions are thought to neutralize fungal spores as they pass through the termite gut. Also, when the symbiont community is disrupted pathogen susceptibility increases. These recent discoveries have shed light on novel interactions for symbiotic microbes both within the termite host and with pathogenic invaders. Lower termite biology is therefore tightly linked to symbiotic associations and their resulting physiological collaborations.

## Introduction

The close association of lower termites with microbes is fundamental to their biology. For the last century, understanding the intricacies of the relationship between termites and their gut symbionts, i.e., the termite holobiont, has been a major focus of termite research. The majority of this work emphasizes both the complexity and novelty of functions carried out to process lignocellulose within the termite gut (reviewed in Brune, 2014). For decades, termite wood digestion has been a quintessential example of symbiotic collaboration; however, symbionts have also been associated with a myriad of other functions in this system (reviewed in Ohkuma, 2008). For example, in addition to synergistic digestive collaboration, symbionts of lower termites have also been shown to play protective roles against pathogens both *in vivo* and *ex vivo* (Rosengaus et al., 1998, 2014; Chouvenc et al., 2009, 2013). This interaction between the termite symbiotic consortium and potential pathogens adds a layer of interplay within this already-complex microbial community. Here we summarize the diversity and roles symbionts play in lower termites, highlight the broad implications of both topics for understanding termite biology and symbiotic evolution, and emphasize how a holistic approach to studying termite biology is necessary to encompass the impact of this obligate symbiotic association.

Lower termites are distinct from higher-termites in that they form relationships with both eukaryotic and prokaryotic symbionts within their digestive tracts (Eutick et al., 1978). While the diversity, abundance, and functionality of these symbionts fluctuates from species to species, an association with symbionts is ubiquitous and connected with much of the biology of termites. Fundamental defining aspects of lower termites, from eusociality to niche occupation, are impacted by their obligate association with microbes. Disruption of this community impacts termite physiological function, fitness, and survivorship (Cleveland, 1924; Thorne, 1997; Rosengaus et al., 2011b, 2014; Peterson et al., 2015; Sen et al., 2015). Lower termites house protists (unicellular eukaryotes), bacteria, and archaea all within the one-microliter environment of their hindgut, many of which are never found outside of this association. Restricted to their association with termites, these symbionts are exposed to and must tolerate a variety of chemical and biological stressors in the termite gut microenvironment. As the host termite feeds, forages, grows, and encounter pathogens, its symbiota are impacted. Thus, termites cannot be studied without also considering their symbionts. Characterizing and cataloging these microbes poses many challenges because most are unable to be cultured with traditional techniques due to their fastidious nature. This gut microenvironment boasts organismal and metabolic diversity which rivals some of the better studied macro-ecosystems. Approaching the termite holobiont as a fully functional, multifaceted ecosystem allows for concentration on individual species or processes and on the larger collaborative nature of the gut microenvironment.

## Characterizing the Lower Termite Gut Consortium

The key division between lower and higher termite species is the respective nature of their symbiotic partners. While both retain prokaryotic symbionts, lower termites also have flagellated protists living in their guts which is an ancestral trait shared with wood-feeding cockroaches, *Cryptocercus* sp. (Stingl and Brune, 2003; Lo and Eggleton, 2011; Brune and Dietrich, 2015). These protists belong to two groups: the oxymonads and the parabasalids. Originally described as parasites, protists were first found associated with termites over a century ago (Leidy, 1877). Since this original observation, roughly 500 termite-associated protist species have been described (reviewed in Ohkuma and Brune, 2011). As technology advances we are continually able to improve our understanding of the players and complexity of the termite gut community. In fact, new species of protistan symbionts are continually described from lower termite guts (Brugerolle and Bordereau, 2004; Gile et al., 2012; James et al., 2013; Tai et al., 2013; Radek et al., 2014), and the breadth of their diversity is thought to be drastically underestimated in general (Harper et al., 2009; Tai and Keeling, 2013). That being said, lower termites are thought to possess anywhere from a few to a dozen protist species as symbionts that maintain tight phylogenetic associations with their hosts (Tai et al., 2015).

As has happened with protist symbionts, our understanding of the bacterial consortium composition in lower termites is constantly evolving as methodologies and analyses improve. Early estimates from the eastern subterranean termite, *Reticulitermes flavipes*, numbered bacteria per gut in the millions, which seems to be a conservative approximation at best (Schultz and Breznak, 1978). Using culture-independent, cloning based methods, several groups have estimated the guts of lower termite species to contain anywhere from 222–1,318 ribotypes of bacteria (Hongoh et al., 2003a,b; Shinzato et al., 2005; Yang et al., 2005; Fisher et al., 2007). With the onset of next-generation sequence technologies this number has only grown. More recently, the gut lumen content of *R. flavipes* workers was described to contain over 4,761 species-level phylotypes of prokaryotic symbionts, with over 99% being bacteria (Boucias et al., 2013). The majority of these identified phylotypes are unique to the termite gut, having never been reported elsewhere and not having close-relative sequences available in databases. *Coptotermes gestroi* has been estimated to house 1,460 species of bacteria using Illumina technology (Do et al., 2014). These estimates vary for a variety of possible reasons, including local environment, study locus, methodological limitations/caveats, sampling strategy, diet, genetic background, and termite species. While identifying the microbial players within this system is an important step, describing the functions and interplay between them will be equally necessary for understanding termite biology and evolution.

## Symbiotic Collaboration in Termite Digestion and Nutrition

Apart from cataloging symbiont diversity, much of termite research has focused on their associations with the symbiotic microbes which aid in wood digestion. Feeding on this lignin-rich, nitrogen-poor diet requires a suite of enzymes both to catalyze its breakdown and supplement its nutritional deficiencies. Termites and their symbionts complement each other’s capabilities in this way. Termites contribute several highly active enzymes important to this process including endogenous cellulases (β-1, 4-endoglucanase, β-glucosidase) and lignin/phenolic detoxifiers (aldo-keto reductase, laccase, catalase, cytochrome p450s) (Scharf et al., 2010; Zhou et al., 2010; Raychoudhury et al., 2013; Sethi et al., 2013b). Protists in the hindgut of lower termites have been credited with the contribution of several important glycosyl hydrolases (GHFs 5, 7, 45) which aid in cellulolytic activity (Ohtoko et al., 2000; Todaka et al., 2010; Sethi et al., 2013a) and are important in hydrogen cycling (Inoue et al., 2005, 2007). Based on transcriptomic studies, protists possess many more potentially important cellulases (Todaka et al., 2007; Tartar et al., 2009). Also, both the termite host and protist symbionts possess proteases which may be important for utilizing bacteria as sources of nitrogenous compounds (Sethi et al., 2011; Tokuda et al., 2014). Although both protists and bacteria possess many hemicellulases (Inoue et al., 1997; Tartar et al., 2009; Tsukagoshi et al., 2014), termite endogenous cellulases have been shown to have hemicellulase activity as well (Scharf et al., 2010, 2011; Karl and Scharf, 2015). However, despite this apparent hemicellulolytic redundancy, protists, bacteria, and archaea in the hindgut paunch clearly all contribute significantly to the overall efficiency of wood digestion (Peterson et al., 2015).

While protists are mainly responsible for lignocellulolytic activity, the prokaryotic community provides a more diverse subset of services in the termite gut. Spirochetes, the most conspicuous bacterial group in lower termite guts, are capable of diverse metabolic processes including acetogenesis, nitrogen fixation, and degradation of lignin phenolics (Lilburn et al., 2001; Graber and Breznak, 2004; Lucey and Leadbetter, 2014). The isolation and maintenance of pure cultures of several species of spirochetes from lower termite guts has been a powerful tool for describing their metabolic capabilities and collaborative potential within the community as a whole (Leadbetter et al., 1999; Lilburn et al., 2001; Salmassi and Leadbetter, 2003; Graber and Breznak, 2004, 2005; Graber et al., 2004; Dröge et al., 2006; Rosenthal et al., 2011).

Another major component of lower termite microbiota are the bacteria which are intimately associated with gut flagellates as intracellular endosymbionts (Stingl et al., 2005; Noda et al., 2009). There are four phyla of bacterial endosymbionts found within protist cells: Elusimicrobia, Bacteroidetes, Proteobacteria, and Actinobacteria (Hara et al., 2004; Noda et al., 2005; Stingl et al., 2005; Strassert et al., 2012). These groups have been found to ferment glucose, synthesize amino acids, produce cofactors, fix nitrogen, and recycle nitrogenous wastes (Noda et al., 2007; Hongoh et al., 2008a,b; Ohkuma and Brune, 2011; Strassert et al., 2012; Zheng et al., 2015). *Methanobrevibacter*, a methanogenic archaeal genus common across termite-associated flagellates, contribute methane to the gut environment using hydrogen that is present in copious amounts in the gut lumen as a product of cellulose metabolism (Shinzato et al., 1999; Tokura et al., 2000; Hara et al., 2004; Hongoh and Ohkuma, 2011). This adds another level of complexity to termite gut ecology by creating a tripartite symbiosis: *prokaryotes within protozoa within termites*.

Apart from archaea associated with termite gut flagellates, representative Methanobacteriaceae are also associated with the microaerobic termite gut lining (Leadbetter and Breznak, 1996; Ohkuma et al., 1999; Brune, 2011). Together with the flagellate endosymbiota, the large amount of methane created by termite digestion can be attributed to archaea which are typically associated with the hindgut lining (Brune, 2011; Hongoh and Ohkuma, 2011). In sum, the microbes present in lower termite guts comprise a diverse ecosystem capable of nitrogen cycling, carbohydrate metabolism, methanogenesis, amino acid biosynthesis, hydrogen turnover, and consequently, complementing deficiencies of the host.

In addition to the contributions of individual organisms, the host fraction (foregut, midgut, and salivary glands) and the symbiont fraction (hindgut) of the termite digestive system have been shown to work synergistically (Scharf et al., 2011). While both fractions have lignocellulolytic activity, combining protein extracts from both the host and symbiont fractions results in more sugar release *in vitro* than the sum of the parts. Additionally, recombinant host and symbiont enzymes have been shown to work efficiently *in vitro* to liberate glucose and pentose sugars from wood (Sethi et al., 2013a). Hence, wood digestion is truly the result of successful collaboration between termites and their hindgut symbionts. This collaborative physiological functionality is a driver in termite success and niche occupation, and it should continue to be a major focus to understand termite holobiont biology and ecology as we go forward.

## Symbiont–Pathogen Interplay

Social living and foraging in microbe-rich environments puts termite workers at risk to encounter pathogens and creates the potential for epizootic events within termite colonies. Though the relationship between termites and their symbionts is often perceived to be purely nutritional, there is growing evidence that gut microbiota have infection-buffering potential. However, termites also have evolved complex hygienic behaviors to mitigate the spread and persistence of pathogenic agents (i.e., fungal conidia) within colonies (Rosengaus et al., 1998; Rosengaus et al., 2011a; Gao et al., 2012). Termites have been frequently observed to auto- and allogroom conidia from the bodies of themselves and nestmates. Passage through the alimentary canal and symbiont-filled hindgut effectively neutralizes fungal conidia (Chouvenc et al., 2009). Termites with perturbed gut microbiota, by oxygenation or chemical means, display a marked increase in susceptibility to fungal pathogens such as *Metarhizium anisopliae* and *Beauveria bassiana* (Boucias et al., 1996; Ramakrishnan et al., 1999; Rosengaus et al., 2014; Sen et al., 2015). One biochemical mechanism has been linked to this anti-fungal gut phenomenon in the form of symbiont-derived β-1, 3-glucanase activity (most likely protist) that is able to act on fungi and prevent their germination (Rosengaus et al., 2014). Similarly, the inhibition of this antifungal enzyme activity, β-1, 3-glucanase, results in a marked increase in termite susceptibility to a variety of pathogens (Bulmer et al., 2009) and is conserved evolutionarily from woodroaches to termites (Bulmer et al., 2012).

As mentioned above, grooming and hygienic behavior play an important role in termite immunity. Termites also participate in proctodeal trophallaxis as a means to replenish symbionts, nutrients, and chemical signals amongst individuals in the colony (Suarez and Thorne, 2000; Machida et al., 2001). This is another means by which symbionts and potential pathogens may interact, but it does not seem to play an important role in immune priming (Mirabito and Rosengaus, 2016).

Lastly, outside of the termite body, symbiotic bacteria provide additional protection. Termites build elaborate nest structures from fecal material to house their colonies. As with hindgut populations, these nest materials contain varying degrees of microbial abundance and richness dependent upon the species of termite (Rosengaus et al., 2003). This material contains diverse kinds of bacteria but has comparatively less fungus (Rosengaus et al., 2003; Manjula et al., 2015). The nests of one species of subterranean termite, *Coptotermes formosanus*, are commonly laden with symbiotic Actinobacteria demonstrated to have antifungal activity *ex vivo* in nest walls (Chouvenc et al., 2013). This finding extends symbiont-mediated protection from the termite gut outside into the nest material, in at least one species.

## Concluding Remarks

Lower termite symbioses with microorganisms are unmistakably integral to termite biology. Hindgut microbial communities are tightly linked with termite digestion of wood and play important roles in supplementing this nutrient-poor food source. Symbionts catalyze reactions involved in the breakdown of all three major components of wood (cellulose, hemicellulose, and lignin phenolics) and supplement this diet by synthesizing other important nutrients. However, outside of the classic role for termite symbionts in digestion and nutrition, there is increasing recognition that they buffer the impacts of environmental stressors to their hosts. In particular, both protists and bacteria have been found to provide anti-fungal defenses in lower termites (Chouvenc et al., 2013; Rosengaus et al., 2014). Even fitness is impacted by the interconnectivity between termites and their symbionts (Rosengaus et al., 2011b). Recent discoveries emphasize that despite nearly a century of studying the obligate relationships between lower termites and microbes, there are still many facets of this complex association which are yet to be understood. Lower termites provide an important model for studying persistent, multi-layer symbioses.

It is also important to consider the role that symbiota play in other animal systems for the purpose of formulating relevant questions to probe, interrogate and eventually understand the termite holobiont. Recent discoveries in other models highlight microbiota as playing more active roles in host physiology, development, and behavior. These roles extend further than the bounds of the intestinal walls, affecting a range of processes from immune system development/maturation to mood and pain perception (Sommer and Backhed, 2013). The broad influence of gut microbiota found in these other systems can serve as an excellent guide to generate hypotheses for testing in the termite system.

Moving forward, based on recent and emerging trends, it will be imperative to consider all components of the termite holobiont when investigating aspects of termite biology. Understanding the role of symbiotic microbes in the physiological processes of digestion and immunity represent some of the first steps toward a better understanding the broader functionality of the lower termite consortium. Viewing any of these interactions within the termite holobiont as discrete may be an oversimplification. However, as methodologies and analyses advance, our ability to understand the functions of the consortium as a whole will continue to improve, as will our understanding of the roles of individual taxa in the system, and collaborations between host and symbiota. Efforts to characterize the holobiont in the presence and absence of stressors, both biotic and abiotic, using comprehensive omics-based approaches are likely to be major hypothesis-generating endeavors. However, the key to doing this successfully will involve careful sample preparation and carefully constructed analysis pipelines to limit taxonomic biases whenever possible. These big data approaches will in turn become a springboard into understanding symbiotic association, trends and commonalities, which may help to begin building models for the compartmentalization, complementation, and collaboration between lower termites and their symbiota.

Understanding the extent, bounds, and ramifications of these associations will be necessary to move toward a fuller appreciation of lower termite biology. Ultimately, studying the collective function and interplay between all members of this symbiosis in response to environmental challenges and in periods of stasis will shed light both on the micro-ecosystem that is a termite gut and the super-organism that is a termite colony.

## Author Contributions

BP and MS developed, wrote, and revised the ideas and content presented in this manuscript. Both BP and MS approve the publishing of this manuscript and take responsibility for all of its contents.

## Conflict of Interest Statement

The authors declare that the research was conducted in the absence of any commercial or financial relationships that could be construed as a potential conflict of interest.
